# The Relationship Between Plasma Fibrinogen Levels and the Severity of Diabetic Foot Ulcers in Diabetic Patients

**DOI:** 10.7759/cureus.81118

**Published:** 2025-03-24

**Authors:** Zahir Khan, Shah Zeb, Ashraf, Rumman, Amjad Ali, Fazal Aleem, Fatima Omair

**Affiliations:** 1 Orthopaedic Surgery, Medical Teaching Institution (MTI) Mardan Medical Complex and Bacha Khan Medical College, Mardan, PAK; 2 Internal Medicine, Medical Teaching Institution (MTI) Mardan Medical Complex and Bacha Khan Medical College, Mardan, PAK; 3 Research and Development, Pro-Gene Diagnostics and Research Laboratory, Mardan, PAK; 4 Pulmonary, Medical Teaching Institution (MTI) Mardan Medical Complex (MMC) Teaching Hospital, Mardan, PAK; 5 Pharmacovigilance/Active Drug Safety Monitoring and Management, Association for Community Development, Peshawar, PAK; 6 Department of Medicine, Bacha Khan Medical College, Mardan, PAK; 7 Nursing, Mardan Medical Complex Teaching Hospital, Mardan, PAK; 8 Biochemistry, Pro-Gene Diagnostics and Research Laboratory, Mardan, PAK

**Keywords:** diabetic foot ulcers (dfus), fibrinogen levels, plasma fibrinogen, vascular impairment, wagner classification

## Abstract

Background

Diabetic foot ulcers (DFUs) are a common and debilitating complication of diabetes mellitus, often leading to hospitalization, amputation, and reduced quality of life. Monitoring biomarkers that reflect inflammatory processes can be crucial for assessing DFU severity and guiding treatment. This study explores the relationship between plasma fibrinogen levels and DFU severity, along with its association with various clinical and inflammatory biomarkers.

Objective

To assess the relationship between plasma fibrinogen levels and DFU severity, vascular health, infection risk, and other biomarkers in diabetic patients, with the aim of improving the prediction and management of DFU outcomes.

Methods

A cross-sectional, observational study was conducted at Mardan Medical Complex from June 2024 to January 2025, involving 93 diabetic patients with active DFUs. The severity of DFUs was classified using the Wagner system, and vascular health was assessed using the Ankle-Brachial Index (ABI) and Doppler ultrasound. Plasma fibrinogen levels, along with other biomarkers such as C-reactive protein (CRP), erythrocyte sedimentation rate (ESR), and fasting blood glucose, were measured through fasting blood samples. Data analysis included statistical tests such as Kruskal-Wallis, Mann-Whitney, ANOVA, Decision Trees, Box Plots, Violin Plot, Histograms, and Regression models to explore the associations between fibrinogen levels and DFU severity, vascular impairment, and inflammatory markers. Significance was set at p < 0.05.

Results

The study examined 93 diabetic patients (mean age: 59.02 ± 7.86 years), comprising 49 males (52.68%) and 44 females (46.32%). Among the participants, 28 (30.11%) presented with severe ulcers (Wagner Grade 5), and 53 (56.99%) exhibited critical ischemia. Bacterial infections were identified in 71 (76.34%) of the patients. The mean plasma fibrinogen level was significantly elevated at 681 ± 160 mg/dL, surpassing the normal range. Plasma fibrinogen levels increased with ulcer severity, with medians of 503.51 mg/dL for Wagner Grade 2, 623.45 mg/dL for grade 3, 627.32 mg/dL for grade 4, and 720.77 mg/dL for grade 5 ulcers. Higher fibrinogen levels were also associated with greater ulcer depth (p = 0.046). Additionally, vascular impairment was significantly correlated with fibrinogen levels, with non-palpable pedal pulses and severe peripheral arterial disease showing associations with elevated fibrinogen levels (p = 0.0083 and p = 0.0478, respectively). Furthermore, fibrinogen levels were positively correlated with CRP (r = 0.50) and with comorbidities such as hypertension in 66 (70.97%) patients and chronic kidney disease in 20 (21.51%) patients.

Conclusion

Plasma fibrinogen levels are strongly associated with DFU severity and could serve as an effective biomarker for predicting disease progression. Monitoring fibrinogen, along with other biomarkers, may help clinicians stratify patients based on their risk of complications and guide more targeted treatment strategies.

## Introduction

Diabetic foot ulcers (DFUs) are one of the most debilitating complications of diabetes mellitus (DM), resulting from a complex interplay of risk factors such as peripheral neuropathy, compromised blood supply, infection, and inflammation [1]. Recent estimates suggest that approximately 15% of diabetic patients will experience a foot ulcer during their lifetime, with hospitalizations due to DFUs significantly impacting healthcare systems [2,3]. Importantly, DFUs can result in severe outcomes, including limb amputations, which affect nearly 85% of patients with a history of non-healing ulcers [4].

Increasing evidence suggests that plasma fibrinogen levels may be closely linked to the severity of DFUs. Fibrinogen, an acute-phase glycoprotein involved in blood coagulation, is known to rise during inflammatory states, making it a valuable biomarker [5]. Several studies indicate that diabetic patients with advanced DFUs often exhibit elevated fibrinogen levels compared to those with milder forms or no ulceration at all [6,7]. For instance, a study reported significantly higher mean fibrinogen levels associated with advanced stages of ulceration as classified by the Wagner system, highlighting fibrinogen's potential role as a biomarker for disease severity [6]. Moreover, the presence of fibrinogen can disrupt normal wound-healing processes, contributing to delayed repair and chronic ulceration [5].

The pro-inflammatory state of diabetes associated with elevated fibrinogen can potentially exacerbate DFU severity through various mechanisms. Higher fibrinogen concentrations may result in increased blood viscosity, leading to vascular occlusion and ultimately impaired healing [8]. Additionally, the inflammatory response, facilitated by fibrinogen, can create a hostile environment for wound healing, characterized by cellular hypoxia and nutrient deprivation [5]. Chronic inflammation present in DFUs involves a range of biomarkers, including fibrinogen, which could play a critical role in both the chronicity and severity of such wounds [6].

Furthermore, some studies have established correlations between fibrinogen levels and common risk factors associated with DFUs, such as metabolic syndrome, cardiovascular diseases, and peripheral artery disease, which are prevalent in diabetic populations [9]. Elevated fibrinogen levels have been shown to correlate with poor glycemic control and increased risk of microvascular complications, further substantiating the inflammatory pathway linking fibrinogen to DFU severity [5.9].

In clinical practice, incorporating plasma fibrinogen level assessments may serve to stratify patients based on ulcer severity, potentially guiding therapeutic interventions and improving outcomes for patients suffering from DFUs [9]. While there is an urgent need for further research to validate these associations and explore potential therapeutic targets, the evidence thus far supports the hypothesis that fibrinogen is a significant biomarker reflecting the severity of diabetic foot conditions [4].

The relationship between plasma fibrinogen levels and the severity of DFUs is an important area for further research. Understanding how fibrinogen contributes to ulcer development and progression can improve our knowledge of diabetic complications and lead to more targeted treatments to prevent severe outcomes. DFUs are a major cause of hospitalization and amputation in diabetic patients, affecting millions globally. Their management is challenging due to slow wound healing, frequent infections, and limited treatment options. Identifying biomarkers like plasma fibrinogen levels could help assess risk, guide treatment decisions, and improve patient outcomes.

This study aims to examine the link between plasma fibrinogen levels and DFU severity, potentially enabling more personalized and effective treatments. Given the significant burden of DFUs on healthcare systems and patients, early identification of prognostic biomarkers could reduce complications, enhance healing, and lower amputation rates in diabetic individuals.

## Materials and methods

Study design and setting

This cross-sectional observational study was conducted at the Department of Orthopedics, Mardan Medical Complex, from June 2024 to January 2025. Diabetic patients with active foot ulcers were recruited using a convenience sampling technique.

Inclusion and exclusion criteria

Adult patients (≥18 years) diagnosed with type 1 or type 2 DM, and presenting with at least one DFU classified according to the Wagner classification system, were included in the study, provided they had stable hemodynamic status and were free from acute cardiovascular events at the time of enrollment. Exclusion criteria consisted of patients with a history of coagulation disorders or those on anticoagulant therapy, individuals with active malignancies, chronic liver disease impacting fibrinogen metabolism, autoimmune diseases, severe infections requiring intensive care, or those undergoing corticosteroid or immunosuppressive therapy. Additionally, patients with non-DFUs resulting from trauma, venous stasis, or arterial causes unrelated to diabetes, as well as those with prior diabetic foot or toe amputations, were excluded.

Sample size calculation

The sample size for this study was determined using GPower 3.1 (The GPower Team, Germany). A moderate effect size (r = 0.30) was selected based on prior research exploring the relationship between plasma fibrinogen levels and DFU severity. With a significance level (α) of 0.05 and a statistical power of 0.80, G*Power calculated that at least 93 participants would be required to detect statistically significant associations. This sample size was considered adequate to assess the relationship between fibrinogen levels and DFU severity, ensuring the study's power to identify meaningful findings. The sample size estimation was also informed by existing literature, which recommended a minimum of 80 participants to achieve statistical significance in detecting variations in plasma fibrinogen levels across different ulcer severities. To account for potential dropouts or missing data, a total of 93 participants were enrolled, and no data were lost during the study, thereby preserving the statistical integrity of the results. [10]

Demographic and clinical data collection

Demographic and clinical data were collected through structured interviews, electronic medical records, and direct clinical examinations. The information recorded included age, gender, body mass index (BMI), smoking status, duration of diabetes (in years), duration of the ulcer, and the presence of any comorbidities.

Diabetic foot ulcer (DFU) assessment

DFU assessment was conducted by evaluating multiple factors, including ulcer depth, location, severity, and the presence of infection. Ulcer severity was classified using the Wagner classification system, which categorizes ulcers according to their severity [11]. The depth of the ulcer was measured in centimeters using a sterile probe to determine the extent of tissue involvement. Ulcer location was documented and categorized as occurring on the toe, heel, sole, dorsum of the foot, or ankle [12]. To assess for infection, culture swabs were obtained from the ulcer site to identify the presence and type of bacterial infection.

Vascular assessment

Vascular assessment was performed to evaluate the presence of peripheral arterial disease (PAD) in patients, beginning with the measurement of the Ankle-Brachial Index (ABI) [13], which compares blood pressure at the ankle and arm using a Doppler ultrasound device. ABI values were interpreted as follows: normal (1.0 - 1.4), indicating no PAD; borderline (0.91 - 0.99), suggesting mild arterial narrowing; abnormal (0.9 - 0.7), indicating mild to moderate PAD; severe PAD (0.69 - 0.4), suggesting significant arterial blockages; and critical ischemia (< 0.4), representing extreme blood flow reduction with a high risk of tissue necrosis and potential amputation. In addition to ABI measurement, vascular Doppler ultrasound was used to further assess vascular health [14], classifying patients as having either moderate vascular impairment or severe vascular disease, with critical ischemia indicating severe blood flow reduction that can lead to serious complications. An ABI value of less than 0.9 was considered indicative of PAD, prompting further clinical evaluation.

Laboratory investigations

Laboratory investigations were conducted to assess various biomarkers related to systemic inflammation, metabolic status, and hematological parameters in the participants. Fasting venous blood samples were collected into citrate tubes, centrifuged to separate plasma, and then analyzed to measure plasma fibrinogen levels using the Clauss fibrinogen assay on a coagulometer (Helena Bioscience C-series) [15], which quantifies clot formation in response to thrombin addition. The reference range for normal fibrinogen levels was considered to be 200-400 mg/dL. In addition to fibrinogen, several other biomarkers were measured to evaluate the participants' overall health. Inflammatory markers included C-reactive protein (CRP), quantified using a turbidimetric immunoassay on the Genrui PA 50, and the erythrocyte sedimentation rate (ESR), assessed by the Westergren method. Metabolic markers included HbA1c levels, measured using the Roche Cobas e411, fasting blood glucose, quantified by the enzymatic glucose oxidase-peroxidase method, and serum albumin, assessed using the colorimetric bromocresol green method on the Roche Cobas C111. Additionally, serum creatinine levels and blood urea nitrogen (BUN) were measured to assess kidney function, also using the Roche Cobas C111. A complete blood count (CBC) was also performed, providing data on white blood cell (WBC) count, neutrophils, lymphocytes, monocytes, eosinophils, basophils, hemoglobin levels, and platelet count using the Horiba Yumizen H500 hematology analyzer.

Statistical analysis

All statistical analyses were performed using IBM SPSS (version 29) and R (version 4.4.2) for comprehensive data analysis and visualization, with Python libraries (Scikit-Learn, Seaborn, Matplotlib) utilized for additional analysis and graphical representation. Prior to conducting statistical tests, data normality was assessed using the Shapiro-Wilk test. Variables with normal distributions were expressed as mean ± standard deviation (SD), while non-normally distributed data were reported as median with interquartile range (IQR). Categorical variables were presented as frequency (n) and percentage (%). To compare plasma fibrinogen levels across different Wagner ulcer grades, the Kruskal-Wallis H-test was employed. The Mann-Whitney U test was used to compare fibrinogen levels between patients with moderate vascular impairment and those with severe vascular disease (critical ischemia). A one-way ANOVA was utilized to examine fibrinogen differences across BMI categories, bacterial infection groups, and ulcer locations. Linear relationships and feature importance analysis were conducted to assess associations between plasma fibrinogen levels and various inflammatory, metabolic, and hematological biomarkers, including CRP, fasting blood glucose, HbA1c, ESR, and WBC count. The strength of these relationships was evaluated through linear regression analysis and the importance of each biomarker in predicting fibrinogen levels was determined using feature importance scores. Correlation coefficients were categorized as weak (0.1-0.3), moderate (0.3-0.6), or strong (0.6-1.0). Decision tree models using the CART (Classification and Regression Tree) algorithm were developed to explore predictive relationships between fibrinogen levels, DFU severity, vascular disease, and ulcer location, with training on 75% of the dataset and validation on 25%. The Gini index was used to identify optimal decision splits at key fibrinogen thresholds for predicting Wagner ulcer grade, vascular impairment, and ulcer location, while feature importance analysis ranked biomarkers by their predictive influence on fibrinogen levels. For data visualization, violin plots were used to compare fibrinogen distributions across DFU severity and vascular disease categories, box plots displayed fibrinogen levels across bacterial infection groups, histograms assessed fibrinogen distribution by ulcer location, and scatter plots illustrated relationships between fibrinogen and inflammatory/metabolic markers. A p-value < 0.05 was considered statistically significant, ensuring clinically and statistically meaningful findings.

Ethical considerations

This study was carried out in accordance with ethical guidelines approved by the Institutional Review Board (IRB) (Approval number: 537/BKMC). Informed consent was obtained from all participants prior to data collection, and confidentiality was ensured by anonymizing personal identifiers. No additional financial or medical burdens were placed on the participants.

## Results

The study analyzed 93 diabetic patients with DFUs, revealing a mean age of 59.02 ± 7.86 years, with a slight male predominance (49 male (52.68%), 44 female (46.32%)). The majority of patients had long-standing diabetes (16.73 ± 7.34 years), with an average ulcer depth of 1.6 ± 0.53 cm. Vascular assessment showed critical ischemia in 53 patients (56.99%), with 39 patients (41.94%) exhibiting weak or absent pedal pulses, indicating significant circulatory impairment. The sole (85 patients, 91.4%) and dorsum of the foot (81 patients, 87.1%) were the most affected ulcer locations, and 28 patients (30.11%) had ulcers classified as severe (Wagner grades 5), highlighting a high risk of complications. Bacterial infections were present in 71 patients (76.34%), with the most common pathogens being *Streptococcus pyogenes* (12 patients, 12.9%), *Klebsiella pneumoniae* (11 patients, 11.83%), and *Proteus mirabilis* (11 patients, 11.83%). The most prevalent comorbidities were hypertension (66 patients, 70.97%), chronic kidney disease (20 patients, 21.51%), and osteomyelitis (10 patients, 10.75%), which further exacerbated ulcer severity (Table 1).

**Table 1:** Demographic and Clinical Profile of Patients with Diabetic Foot Ulcers Data is presented as frequency and percentage or means and standard deviation.

Characteristic	Values
Total no of patients	93 (100)
Age	59.02 ± 7.86
Gender	
Male	49 (52.68)
Female	44 (46.32)
Body mass index (BMI)	24.12 ± 5.14
Smoking History	
No	72 (77.42%)
Yes	21 (22.58%)
Ulcer depth in cm	1.6 ± 0.53
Duration of diabetes	16.73 ± 7.34
Ankle-brachial index (ABI)	0.98 ± 0.25
Vascular Doppler Ultrasound Findings	
Moderate vascular impairment	40 (43.01)
Severe vascular disease (Critical ischemia)	53 (56.99)
Ulcer Location	
Toe	60 (64.52)
Heel	42 (45.16)
Sole	85 (91.40)
Dorsum of foot	81 (87.10)
Ankle	25 (26.88)
Pedal Pulse Status	
Palpable	39 (41.94)
Weak	28 (30.11)
Non-palpable	26 (27.96)
Wagner Classification	
Grade 2	18 (19.35)
Grade 3	08 (8.6)
Grade 4	39 (41.94)
Grade 5	28 (30.11)
Culture Results from Ulcer Swab	
No growth	22 (23.66)
Clostridium species	09 (9.68)
Enterococcus species	09 (9.68)
Escherichia coli	08 (8.60)
Klebsiella pneumoniae	11 (11.83)
Proteus mirabilis	11 (11.83)
Pseudomonas aeruginosa	05 (5.38)
Staphylococcus aureus	06 (6.45)
Streptococcus pyogenes	12 (12.90)
Comorbidities	
Hypertension	66 (70.97)
Osteoporosis	20 (21.51)
Hyperlipidemia	12 (12.90)
Rheumatoid arthritis	12 (12.90)
Peptic ulcer disease	18 (19.35)
Osteomyelitis	10 (10.75)
Chronic kidney disease	20 (21.51)
Coronary artery disease	10 (10.75)
Cellulitis	22 (23.65)

The laboratory findings for the 93 DFU patients revealed several critical biomarkers indicative of poor disease control and systemic inflammation. The mean plasma fibrinogen level was elevated at 681 ± 160, suggesting ongoing inflammation. HbA1c levels were significantly high at 9.18 ± 2.31, reflecting poor long-term glycemic control, while fasting blood glucose was 231.59 ± 126.06, indicating hyperglycemia. Hemoglobin levels were low at 9.39 ± 1.43, suggesting anemia, a common comorbidity in chronic illness. The WBC count was elevated at 19.23 ± 11.92, pointing to an inflammatory response, with neutrophils accounting for 67.36 ± 18.2% of the WBCs, indicative of infection or acute inflammation. CRP was also elevated at 31.21 ± 16.95, further supporting the presence of systemic inflammation. Serum albumin levels were low at 3.04 ± 0.54, which may reflect malnutrition or chronic disease. Renal function markers, including serum creatinine (1.891 ± 1.07) and BUN (70.82 ± 46.21), were also elevated, suggesting renal impairment, which is common in diabetic patients (Table 2).

**Table 2:** Laboratory Findings in Patients with Diabetic Foot Ulcers Data is presented as means and standard deviation.

Biomarkers	Values
Plasma fibrinogen level	681±160
HbA1c level	9.18 ± 2.31
Fasting blood glucose level	231.59 ± 126.06
Hemoglobin level	9.39 ± 1.43
White blood cell (WBC) count	19.23 ± 11.92
Neutrophils count	67.36 ± 18.2
Lymphocytes count	19.58 ± 11.78
Monocytes count	107.6 ± 109.75
Eosinophils count	2.13 ± 1.86
Basophils count	1.03 ± 0.77
Platelet count	44670.41 ± 29586.48
Erythrocyte sedimentation rate	113.71 ± 37.68
C-reactive protein level	31.21 ± 16.95
Serum albumin level	3.04 ± 0.54
Serum creatinine level	1.891 ± 1.07
Blood urea nitrogen	70.82 ± 46.21

**Figure 1:**
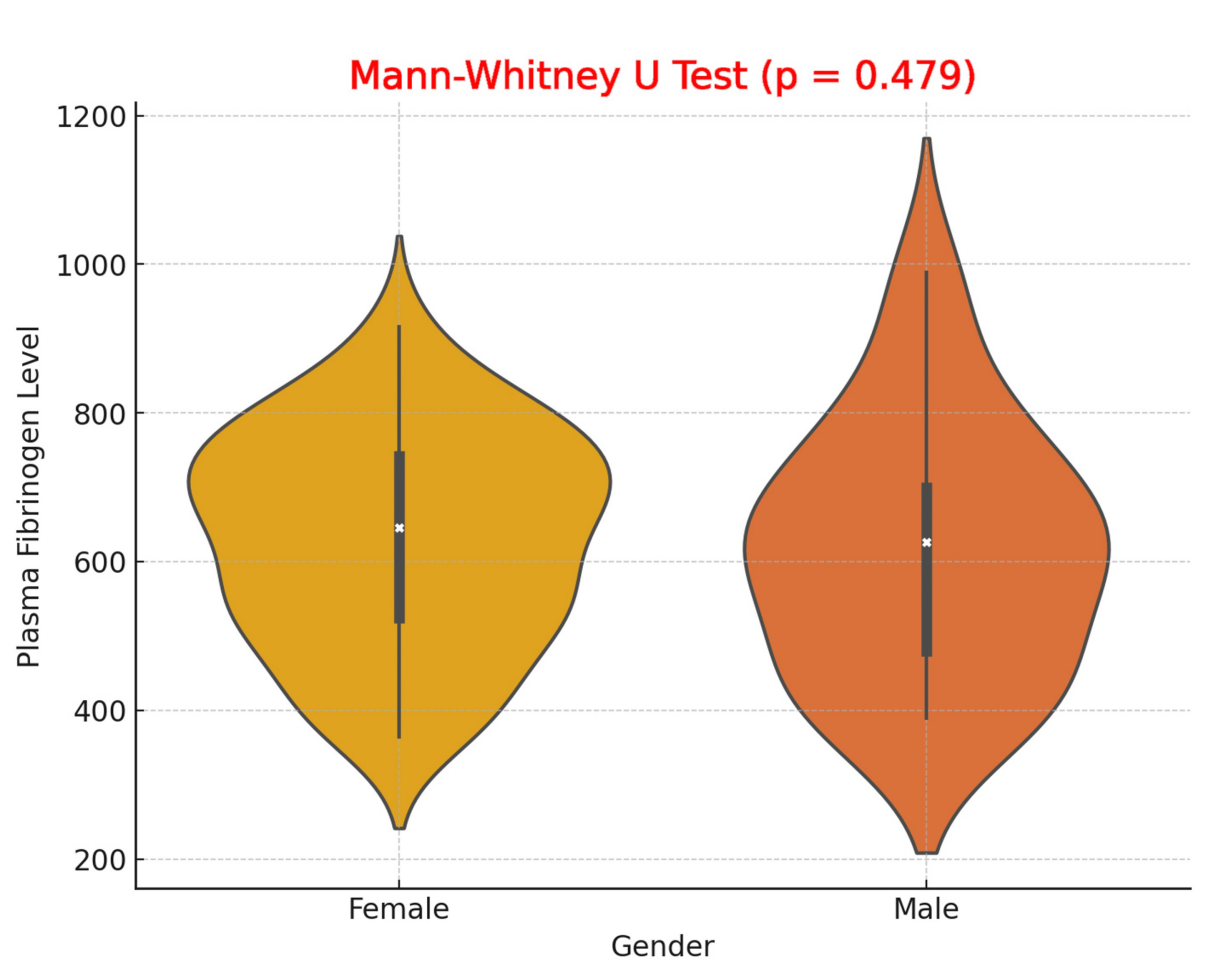
Comparison of Plasma Fibrinogen Levels Between Male and Female Patients with Diabetic Foot Ulcers

The violin plot comparing plasma fibrinogen levels by gender demonstrates that both male and female patients with DFUs exhibit similar ranges of fibrinogen levels, with overlapping distributions. The median fibrinogen levels for both genders are comparable, although females show slightly greater variability. The results of the Mann-Whitney U test yielded a p-value of 0.479, indicating no statistically significant difference in plasma fibrinogen levels between males and females in this study. These findings suggest that gender does not significantly influence fibrinogen levels in patients with DFUs (Figure 1).

**Figure 2:**
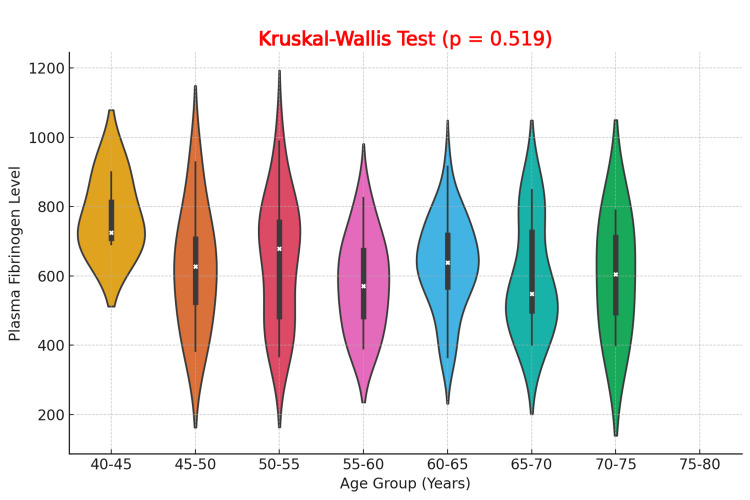
Comparison of Plasma Fibrinogen Levels Across different Age Groups in Diabetic Foot Ulcer Patients

The violin plot comparing plasma fibrinogen levels across different 5-year age groups (ranging from 40 to 80 years) shows similar distributions of fibrinogen levels across all groups. No discernible trend of increasing or decreasing fibrinogen levels with age is observed, as the spread, median, and interquartile range remain consistent across all age groups. The Kruskal-Wallis Test yielded a p-value of 0.519, indicating no statistically significant difference in plasma fibrinogen levels between the age groups. These results suggest that age does not substantially influence plasma fibrinogen levels in this cohort of DFU patients (Figure 2).

**Figure 3:**
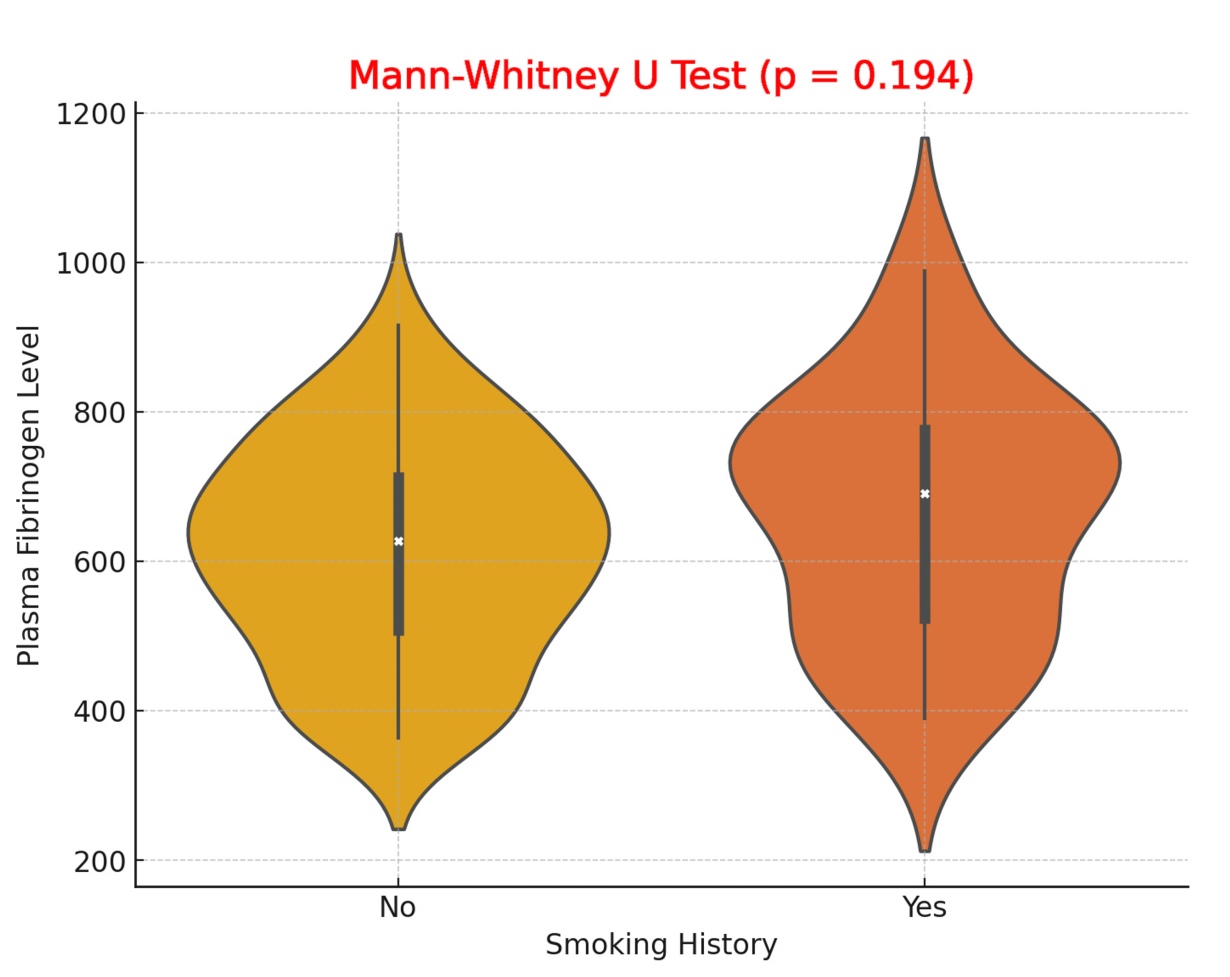
Comparison of Plasma Fibrinogen Levels in Diabetic Foot Ulcer Patients with and without Smoking History

The violin plot comparing plasma fibrinogen levels based on smoking history reveals that while both smokers and non-smokers exhibit a broad range of fibrinogen levels, no significant difference is observed in the distributions between the two groups. The Mann-Whitney U test yielded a p-value of 0.194, indicating that smoking history does not have a statistically significant effect on plasma fibrinogen levels in patients with DFUs. Both smokers and non-smokers have comparable median fibrinogen levels, and the variability in fibrinogen levels is similar across the two groups (Figure 3).

**Figure 4:**
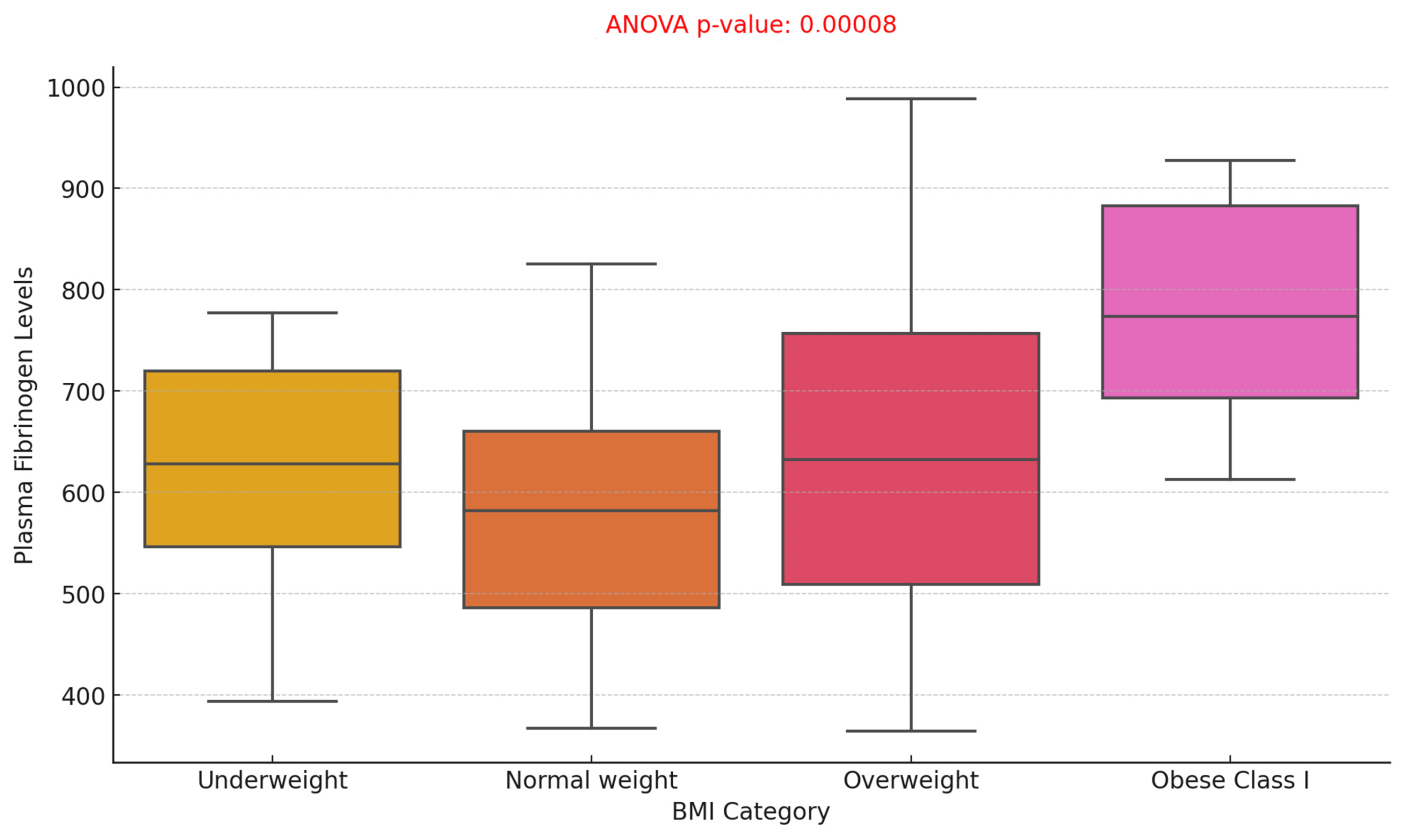
Association Between Plasma Fibrinogen Levels and BMI Categories in Diabetic Foot Ulcer Patients with Fibrinogen Ranges

The box plot illustrating plasma fibrinogen levels across various BMI categories (Underweight, Normal weight, Overweight, and Obese Class I) demonstrated significant variations in fibrinogen levels between groups. The ANOVA p-value of 0.00008 indicates a statistically significant association between BMI and plasma fibrinogen levels. Notably, the fibrinogen levels exhibited an upward trend with increasing BMI categories. These findings suggest that a higher BMI is associated with elevated plasma fibrinogen levels, likely reflecting increased systemic inflammation commonly observed in obesity (Figure 4).

**Figure 5:**
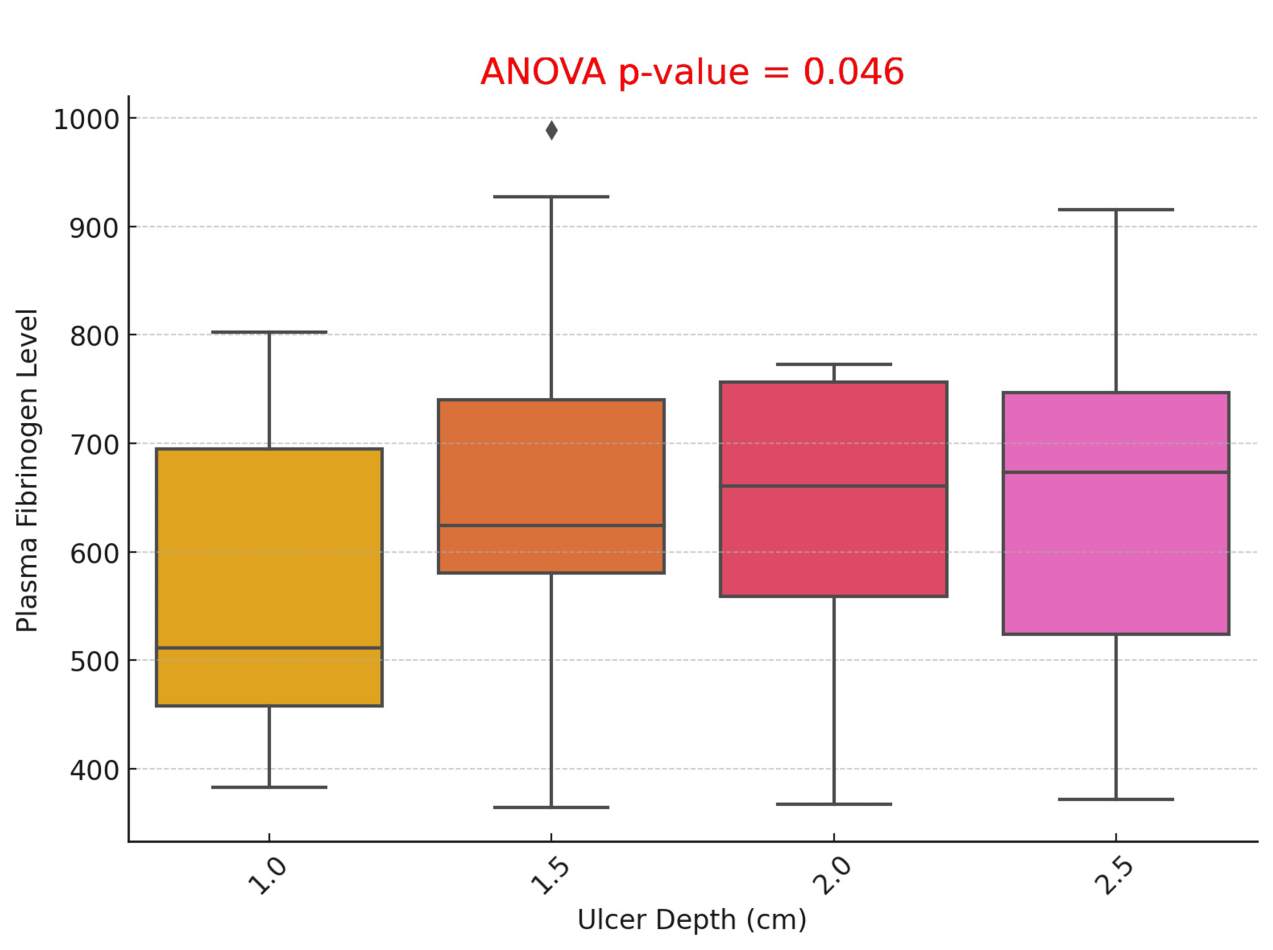
Association Between Plasma Fibrinogen Levels and Ulcer Depth in Diabetic Foot Ulcer Patients

The box plot comparing plasma fibrinogen levels by ulcer depth (1.0 cm, 1.5 cm, 2.0 cm, and 2.5 cm) demonstrates a significant increase in fibrinogen levels with greater ulcer depth, as indicated by an ANOVA p-value of 0.046, confirming a statistically significant relationship. These findings suggest that increased ulcer depth is associated with higher plasma fibrinogen levels, likely reflecting greater inflammation and tissue damage in more severe ulcers (Figure 5).

**Figure 6:**
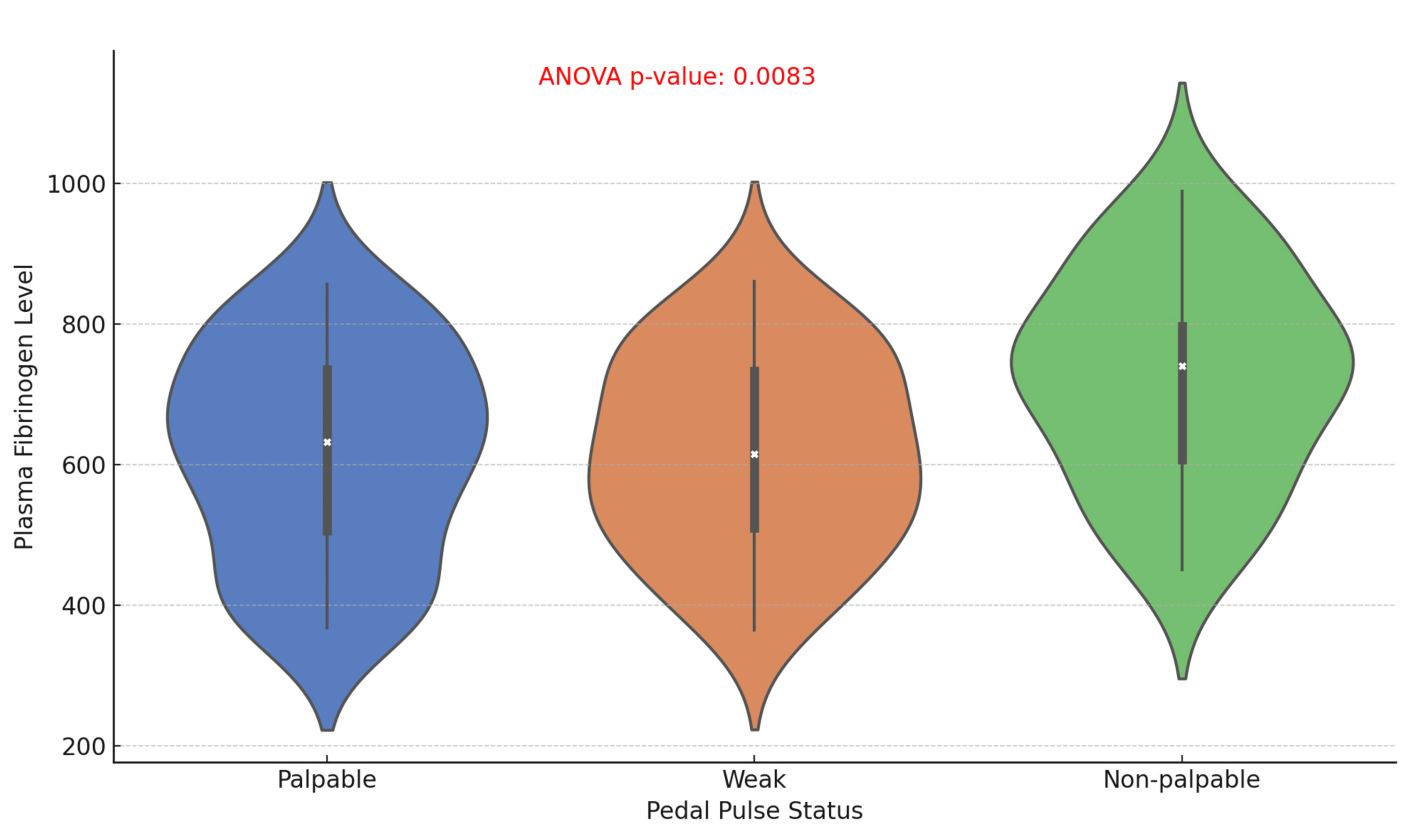
Association Between Plasma Fibrinogen Levels and Pedal Pulse Status in Diabetic Foot Ulcer Patients

The violin plot comparing plasma fibrinogen levels by pedal pulse status (Palpable, Weak, and non-palpable) reveals significant variation in fibrinogen levels, with an ANOVA p-value of 0.0083, indicating a statistically significant relationship. These findings suggest that poorer pedal pulse status, particularly non-palpable pulses, is associated with higher plasma fibrinogen levels, likely reflecting increased vascular impairment and inflammation in these patients (Figure 6).

**Figure 7:**
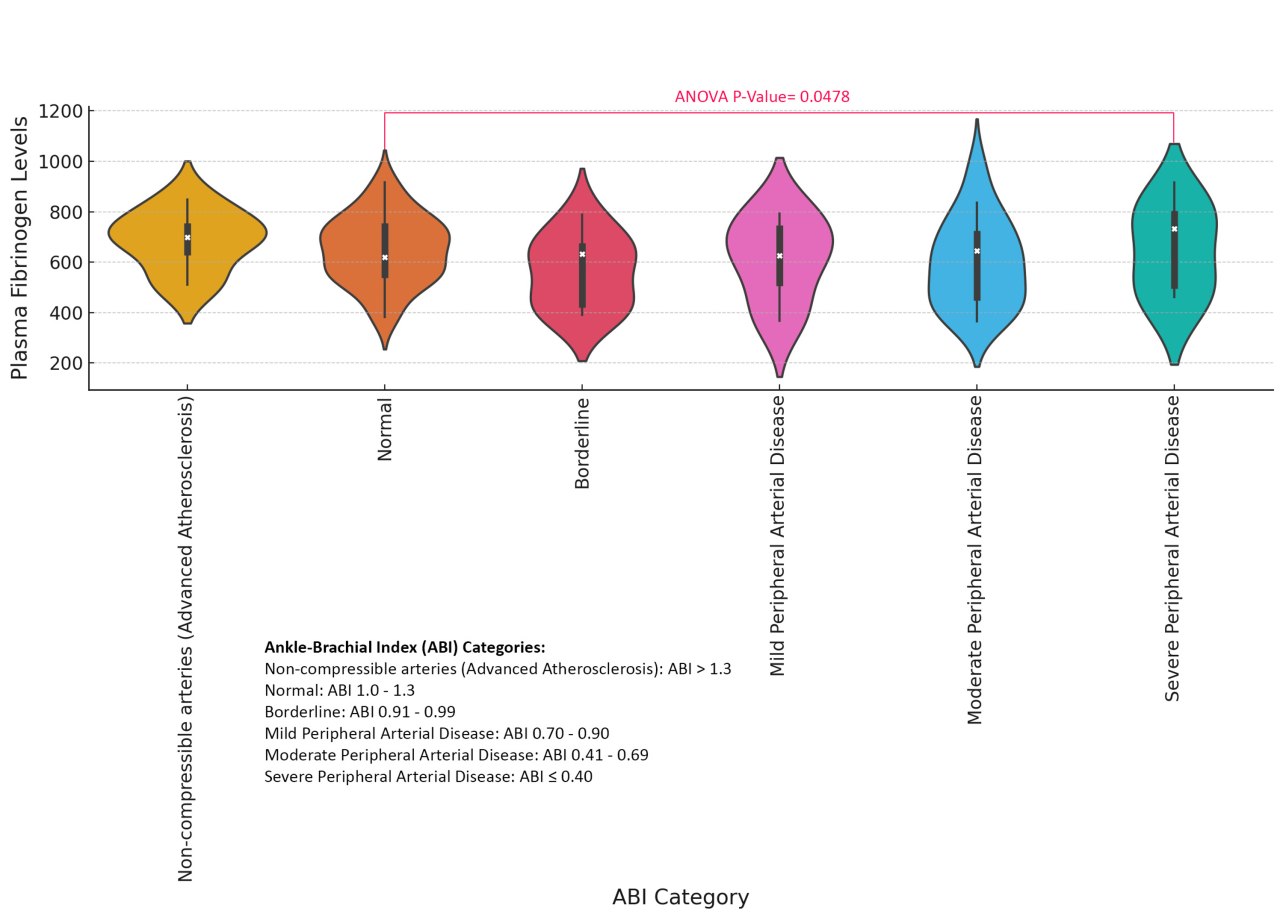
Association Between Plasma Fibrinogen Levels and Ankle-Brachial Index (ABI) Categories in Diabetic Foot Ulcer Patients

The violin plot comparing plasma fibrinogen levels across different Ankle-Brachial Index (ABI) categories (Normal, Borderline, Mild PAD, Moderate PAD, and Severe PAD) reveals significant variation in fibrinogen levels, with an ANOVA p-value of 0.0478, indicating a statistically significant relationship between ABI and fibrinogen levels. These findings suggest that lower ABI values, indicative of worsening PAD, are associated with higher plasma fibrinogen levels, reflecting increased vascular impairment and inflammation (Figure 7).

**Figure 8:**
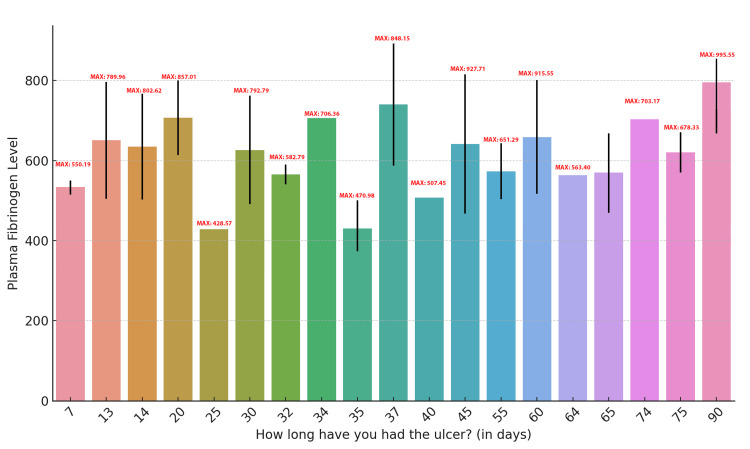
Plasma Fibrinogen Levels Across Different Ulcer Durations in Diabetic Foot Ulcer Patients

The bar chart illustrating plasma fibrinogen levels versus ulcer duration (in days) reveals notable variation in fibrinogen levels across different ulcer durations. The maximum fibrinogen levels for each ulcer duration are as follows: 7 days (maximum = 550.19), 13 days (maximum = 789.96), 14 days (maximum = 802.62), 20 days (maximum = 706.36), 25 days (maximum = 582.79), and 30 days (maximum = 470.98). As ulcer duration increases, maximum fibrinogen values tend to rise, reaching 995.55 at 90 days. The chart also includes error bars, which reflect the variability in fibrinogen levels within each ulcer duration group. These findings suggest that plasma fibrinogen levels tend to increase with the duration of the ulcer, potentially indicating heightened systemic inflammation as the wound persists (Figure 8).

**Figure 9:**
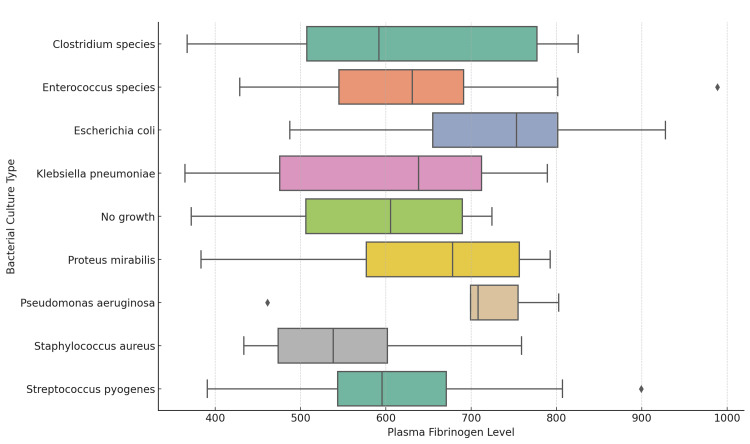
Interaction Between Plasma Fibrinogen Levels and Bacterial Culture Types in Diabetic Foot Ulcer Patients

The box plot illustrating the interaction effect between plasma fibrinogen levels and bacterial culture types reveals that bacterial infections are associated with higher fibrinogen levels compared to the “No growth” category. Infections with bacterial growth show significantly elevated fibrinogen levels, indicating a stronger systemic inflammatory response. In contrast, the “No growth” category is linked to lower fibrinogen levels, suggesting a reduced or absent inflammatory response. These findings highlight that the presence of bacterial growth generally triggers higher fibrinogen levels, while the absence of infection is associated with lower levels (Figure 9).

**Figure 10:**
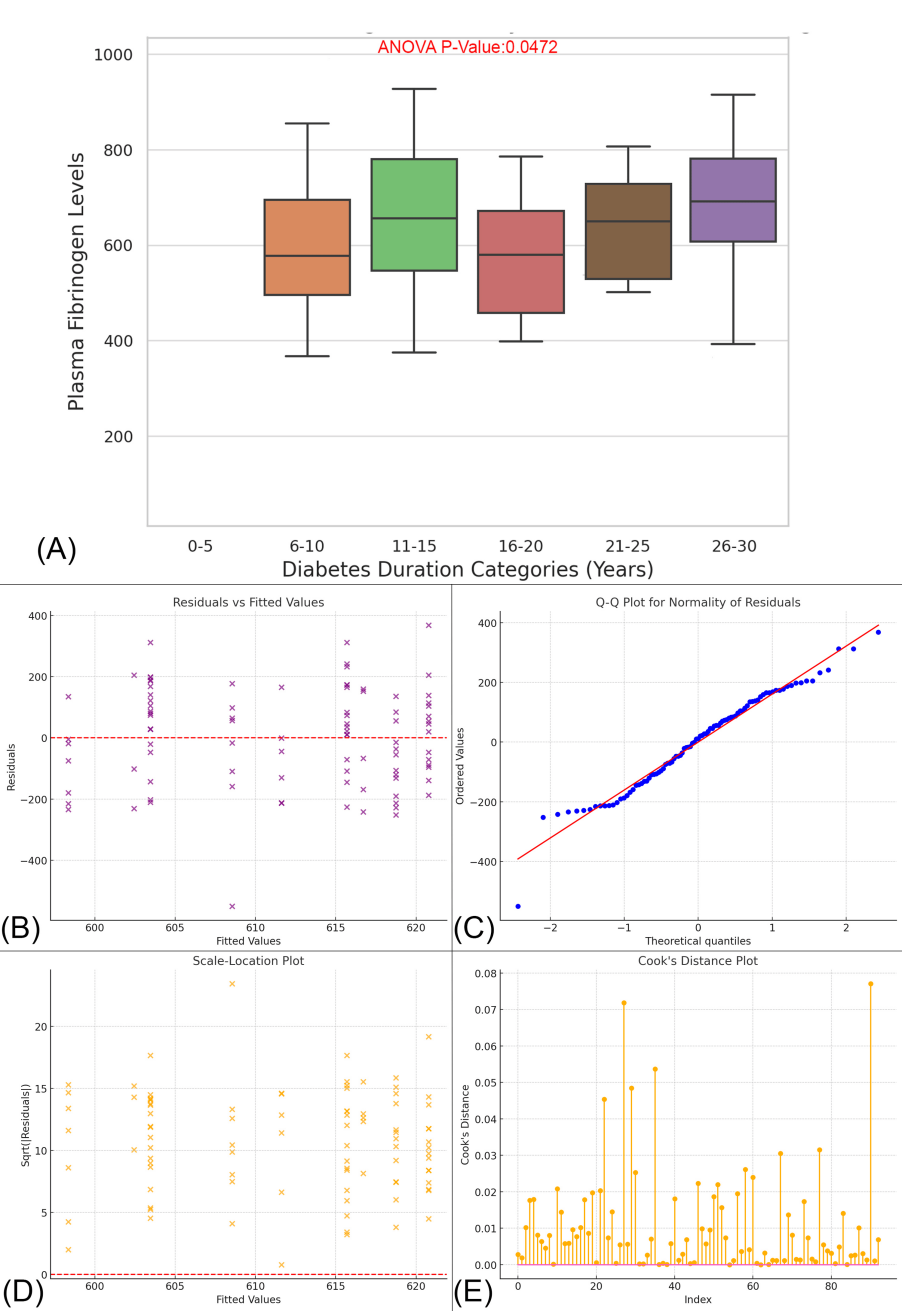
Association Between Plasma Fibrinogen Levels and Diabetes Duration in Diabetic Foot Ulcer Patients (A) Box Plot shows a significant difference in plasma fibrinogen levels across diabetes duration categories (ANOVA p-value = 0.0472). Residuals vs Fitted Values (B): Indicates no obvious pattern, suggesting the model fits the data well; Q-Q Plot (C): The residuals follow a normal distribution, indicating normality; Scale-Location Plot (D): Shows no issue with variance, confirming homoscedasticity. Cook’s Distance Plot (E): Identifies a few influential points that could affect the regression model.

The box plot illustrates the relationship between plasma fibrinogen levels and diabetes duration, divided into six categories ranging from 0-5 to 26-30 years. The data reveals a clear upward trend in plasma fibrinogen levels as diabetes duration increases, particularly in the 16-20, 21-25-, and 26-30-years categories, where median fibrinogen levels progressively rise. Furthermore, the variability in fibrinogen levels increases with longer diabetes duration, with the 26-30 years category exhibiting the widest spread. The ANOVA p-value of 0.0472 indicates a statistically significant difference in fibrinogen levels across the diabetes duration groups, suggesting that longer diabetes duration is associated with higher plasma fibrinogen levels. Diagnostic plots for the statistical model demonstrate a good fit, with normally distributed residuals, constant variance, and no influential points, reinforcing the robustness of the analysis (Figure 10).

**Figure 11:**
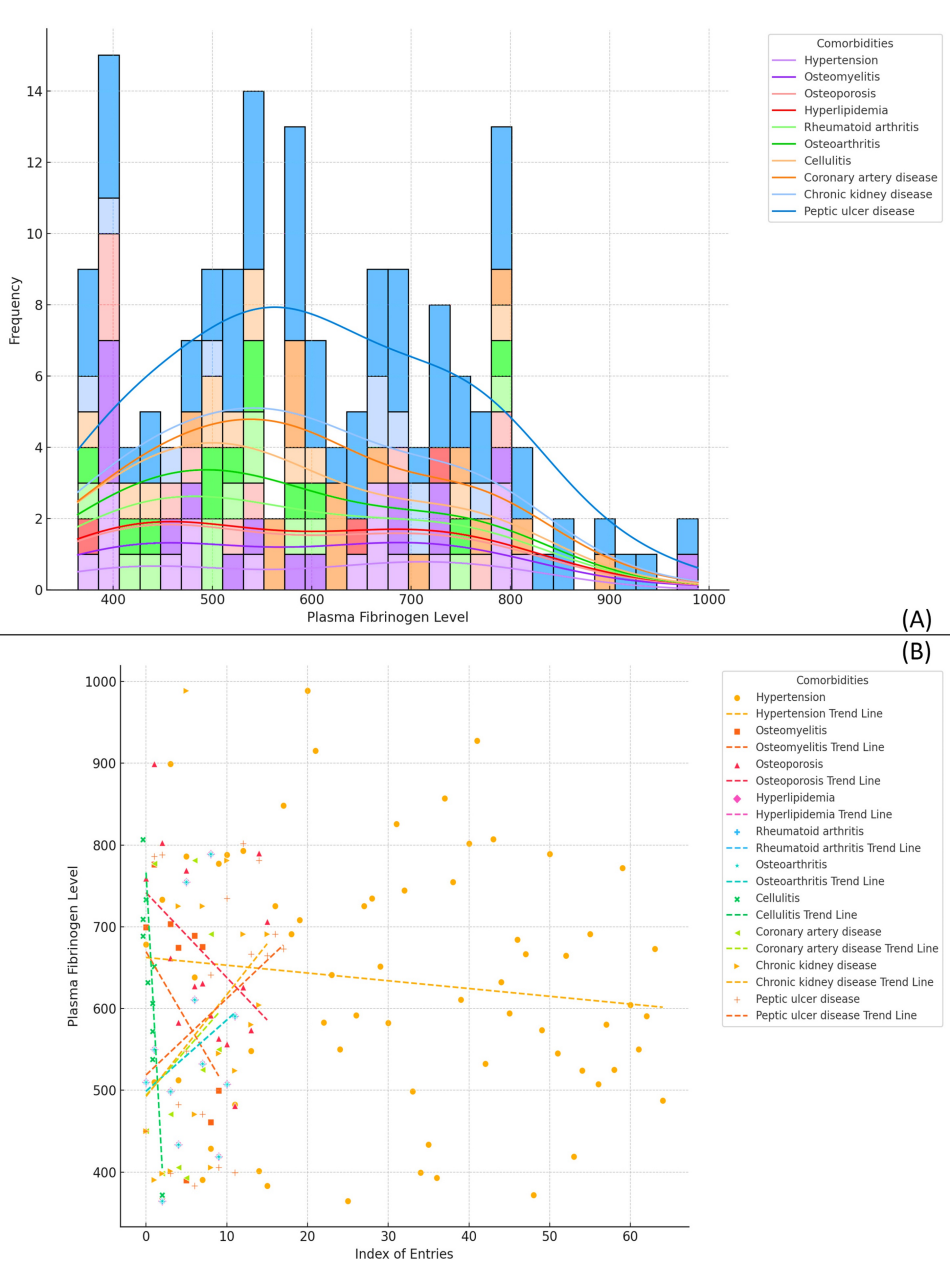
Plasma Fibrinogen Levels in Diabetic Foot Ulcer Patients by Comorbidity Type (A) The distribution of plasma fibrinogen levels across different comorbidity types is shown using a histogram with stacked bars. Each comorbidity (e.g., hypertension, osteomyelitis, osteoporosis) has a distinct distribution pattern, with smooth curves indicating the trend within each group. (B) A scatter plot of plasma fibrinogen levels by comorbidity type, with trend lines for each condition. These trend lines show the general direction of fibrinogen levels across the different comorbidities, helping to identify potential relationships between comorbidities and fibrinogen levels.

The plots illustrate the distribution of plasma fibrinogen levels across various comorbidity types in the study population. Panel A shows the frequency distribution of fibrinogen levels for different comorbidities, with distinct patterns for each comorbidity type. It is evident that certain comorbidities, such as hypertension and chronic kidney disease, tend to show higher fibrinogen levels, particularly in the 600 to 800 range. Conversely, other conditions like rheumatoid arthritis and peptic ulcer disease exhibit a more evenly distributed or lower range of fibrinogen levels. Panel B provides scatter plots with trend lines for each comorbidity type, highlighting the relationships between comorbidity and fibrinogen levels. The trend lines indicate a generally positive association between fibrinogen levels and conditions like osteomyelitis and hypertension, while conditions such as osteoarthritis show a weaker or negative trend. Overall, the analysis suggests that patients with certain comorbidities, especially those linked to chronic inflammation, tend to have higher plasma fibrinogen levels, reflecting the systemic inflammatory burden associated with these conditions (Figure 11).

**Figure 12:**
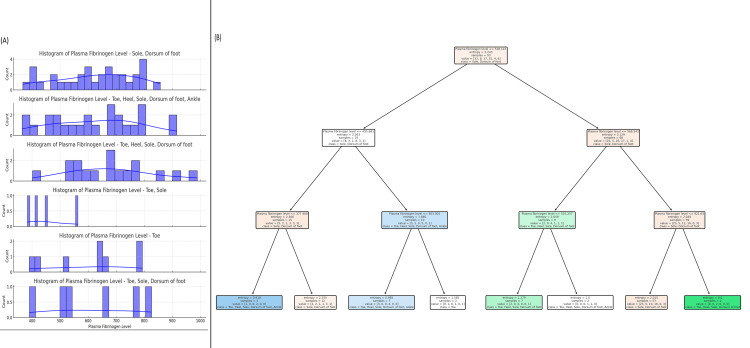
Predicting Ulcer Location in Diabetic Foot Ulcer Patients Using Plasma Fibrinogen Levels: A Decision Tree Approach (A) Histograms of plasma fibrinogen levels for various ulcer locations on the body, including the sole, dorsum of the foot, heel, ankle, and toe. Each histogram shows the distribution of fibrinogen levels, highlighting the variations in plasma fibrinogen across different ulcer locations. (B) A decision tree used for predicting the ulcer location based on plasma fibrinogen levels. The tree splits the data at different fibrinogen level thresholds, providing classifications for ulcer locations (e.g., sole, dorsum of foot, toe, heel, ankle) at each node. The entropy values indicate the purity of the splits, with lower entropy values indicating better classification accuracy. splits the data at different fibrinogen level thresholds, providing classifications for ulcer locations (e.g., sole, dorsum of foot, toe, heel, ankle) at each node. The entropy values indicate the purity of the splits, with lower entropy values indicating better classification accuracy.

The plot displays the relationship between plasma fibrinogen levels and ulcer location, utilizing histograms and a decision tree model. Panel A shows histograms of plasma fibrinogen levels across different ulcer locations, including the sole, dorsum of foot, toe, heel, and combinations of these sites. The distributions reveal the following patterns: for the sole, dorsum of foot, fibrinogen levels range from 400 to 900, with a peak around 700; for the toe, heel, sole, dorsum of foot, the fibrinogen levels range from 400 to 800, with a peak around 600; for the toe, sole, the range is 400 to 850, with a median near 600; for the toe, levels range from 400 to 750, with a median of approximately 600; and for the toe, sole, dorsum of foot, fibrinogen levels range from 400 to 800, with a median around 600. Panel B presents a decision tree model that predicts ulcer location based on fibrinogen levels. The tree demonstrates that plasma fibrinogen levels below 453.661 are associated with ulcers on the sole, dorsum of foot, while higher levels predict ulcers on the toe, heel, sole, dorsum of foot, and ankle. The decision tree model's entropy values indicate that fibrinogen levels are effective in classifying ulcer locations, with the highest accuracy seen in the toe, heel, sole, dorsum of foot, and ankle group. These findings highlight a clear relationship between plasma fibrinogen levels and ulcer location, suggesting that fibrinogen could be a useful biomarker for predicting the site of DFUs (Figure 12).

**Figure 13:**
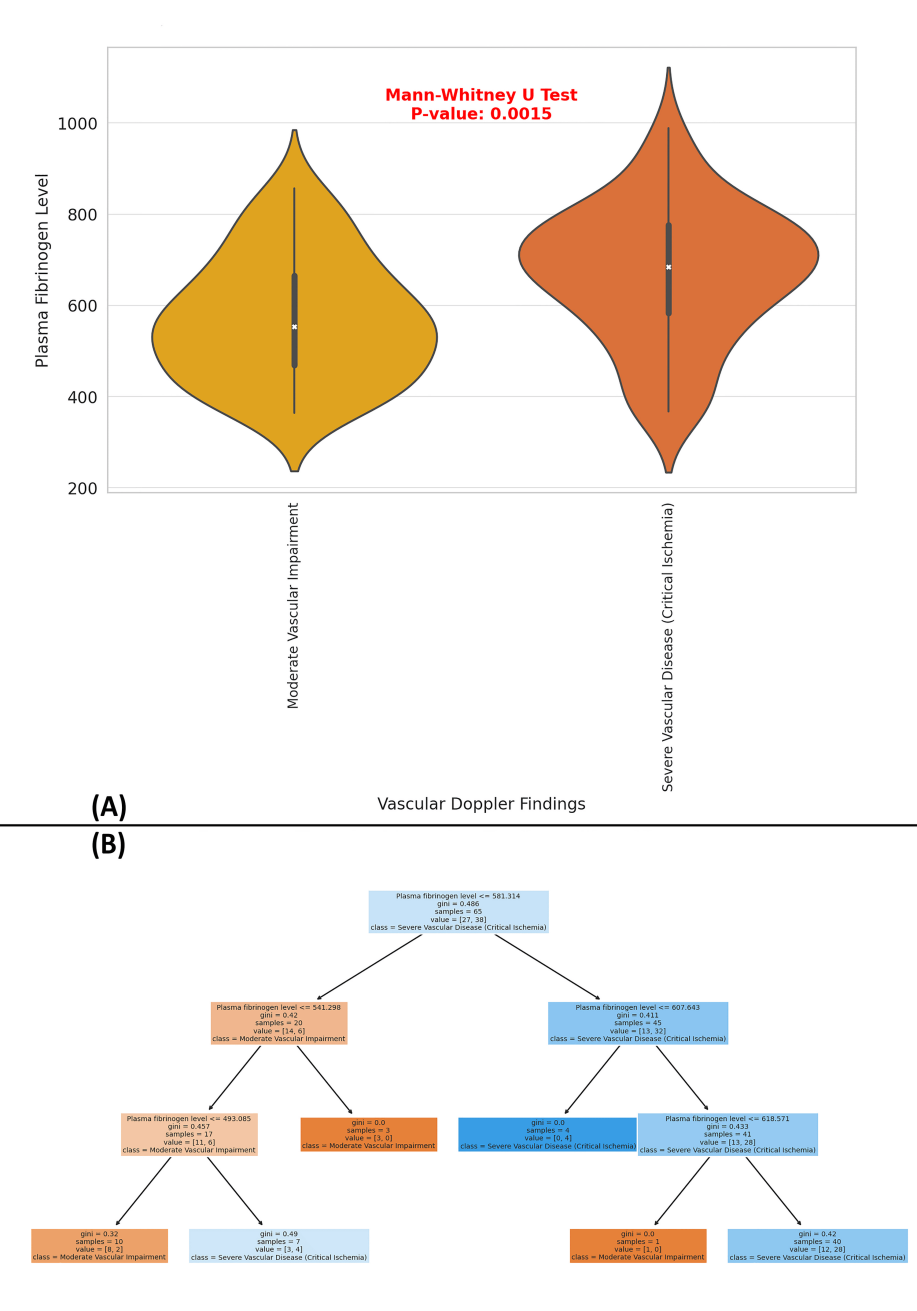
Plasma Fibrinogen Levels as a Predictor of Vascular Disease Severity in Diabetic Foot Ulcer Patients: A Decision Tree Analysis (A) A violin plot showing plasma fibrinogen levels for two vascular conditions: Moderate Vascular Impairment and Severe Vascular Disease (Critical Ischemia). The plot indicates a significant difference between the two groups (p-value = 0.0015). (B) A decision tree classifying vascular findings based on plasma fibrinogen levels. The tree shows how fibrinogen thresholds predict the severity of vascular disease, with lower Gini index values indicating more accurate classifications.

The figure consists of two parts: (A) a violin plot of plasma fibrinogen levels by vascular findings and (B) a decision tree for vascular findings classification. The violin plot compares plasma fibrinogen levels between patients with moderate vascular impairment and those with severe vascular disease (critical ischemia). The Mann-Whitney U test p-value is 0.0015, indicating a statistically significant difference in fibrinogen levels between the two groups. Patients with severe vascular disease exhibit higher median fibrinogen levels (~750 mg/dL) with a wider distribution (ranging from 400 to 1000 mg/dL), while those with moderate vascular impairment have a lower median fibrinogen level (~600 mg/dL) and a more compact distribution (ranging from 300 to 850 mg/dL). The decision tree model further classifies vascular impairment severity using plasma fibrinogen thresholds. The first split occurs at 531.314 mg/dL, where fibrinogen levels ≤ 531.314 mg/dL classify as moderate vascular impairment, and values > 531.314 mg/dL indicate severe vascular disease. Additional splits occur at 541.298 mg/dL, 493.085 mg/dL, 607.643 mg/dL, and 618.571 mg/dL, progressively refining the classification. The Gini values (impurity measure) decrease at each split, confirming improved classification accuracy. Notably, fibrinogen levels above 618.571 mg/dL consistently predict severe vascular disease, while values below 493.085 mg/dL indicate moderate vascular impairment. These findings suggest that higher plasma fibrinogen levels are strongly associated with severe vascular disease (critical ischemia), making fibrinogen a potential biomarker for vascular impairment severity in DFU patients (Figure 13).

**Figure 14:**
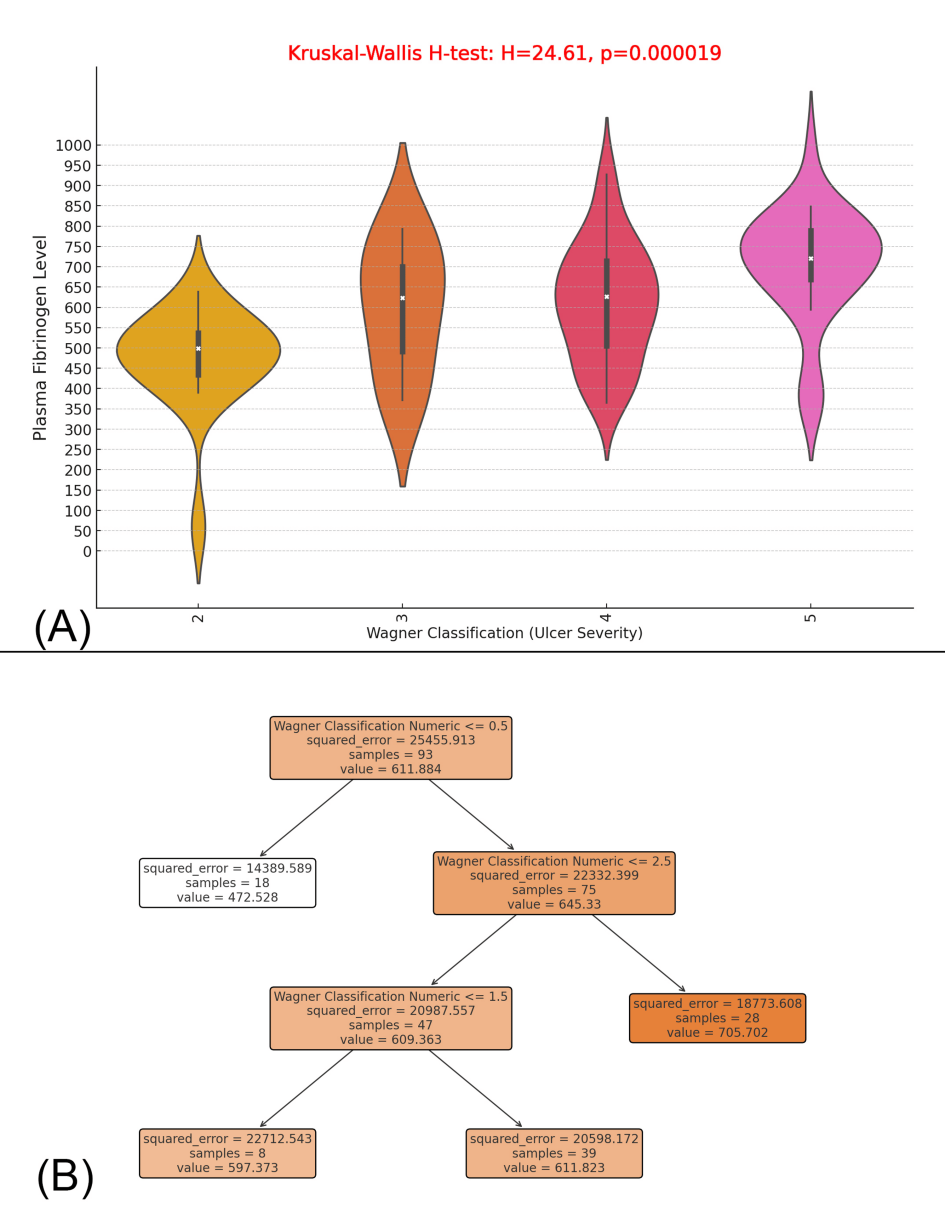
Association Between Plasma Fibrinogen Levels and Diabetic Foot Ulcer Severity: A Violin Plot and Decision Tree Analysis (A) A violin plot showing plasma fibrinogen levels across different Wagner grades (2 to 5) of ulcer severity. The plot demonstrates a significant difference in fibrinogen levels between the ulcer severity grades, with the Kruskal-Wallis H-test showing a result of H = 24.61, p = 0.000019, indicating a statistically significant association between ulcer severity and fibrinogen levels. (B) A decision tree used to predict plasma fibrinogen levels based on Wagner classification (ulcer severity). The tree splits the data based on Wagner Classification Numeric values, with the squared error indicating how well the model predicts fibrinogen levels at each split. The lower the squared error, the better the prediction.

The figure presents (A) a violin plot analyzing plasma fibrinogen levels across different Wagner ulcer severity grades (2 to 5) and (B) a decision tree model predicting fibrinogen levels based on ulcer severity. The Kruskal-Wallis H-test (H = 24.61, p = 0.000019) confirms a statistically significant difference in fibrinogen levels across ulcer grades, with higher Wagner grades (4 and 5) associated with increased fibrinogen levels, reflecting greater inflammation. Wagner Grade 2 ulcers exhibit fibrinogen levels between 458.05 to 554.77 mg/dL (median 503.51 mg/dL), Grade 3 ranges from 491.65 to 700.14 mg/dL (median 623.45 mg/dL), Grade 4 from 528.19 to 714.27 mg/dL (median 627.32 mg/dL), and Grade 5 from 669.30 to 792.31 mg/dL (median 720.77 mg/dL). The decision tree model, with an initial squared error of 25,455.913 across 93 samples, classifies ulcers based on fibrinogen thresholds, splitting at 453.661 mg/dL, further refined at 503.505 mg/dL, 553.257 mg/dL, and 921.603 mg/dL, confirming that higher Wagner grades consistently predict elevated fibrinogen levels. The model identifies that ulcers ≤ Wagner 1.5 have a mean fibrinogen level of 609.363 mg/dL, while ulcers > Wagner 2.5 exhibit 705.702 mg/dL, supporting fibrinogen as a potential biomarker for ulcer severity assessment and progression monitoring in DFUs patients (Figure 14).

**Figure 15:**
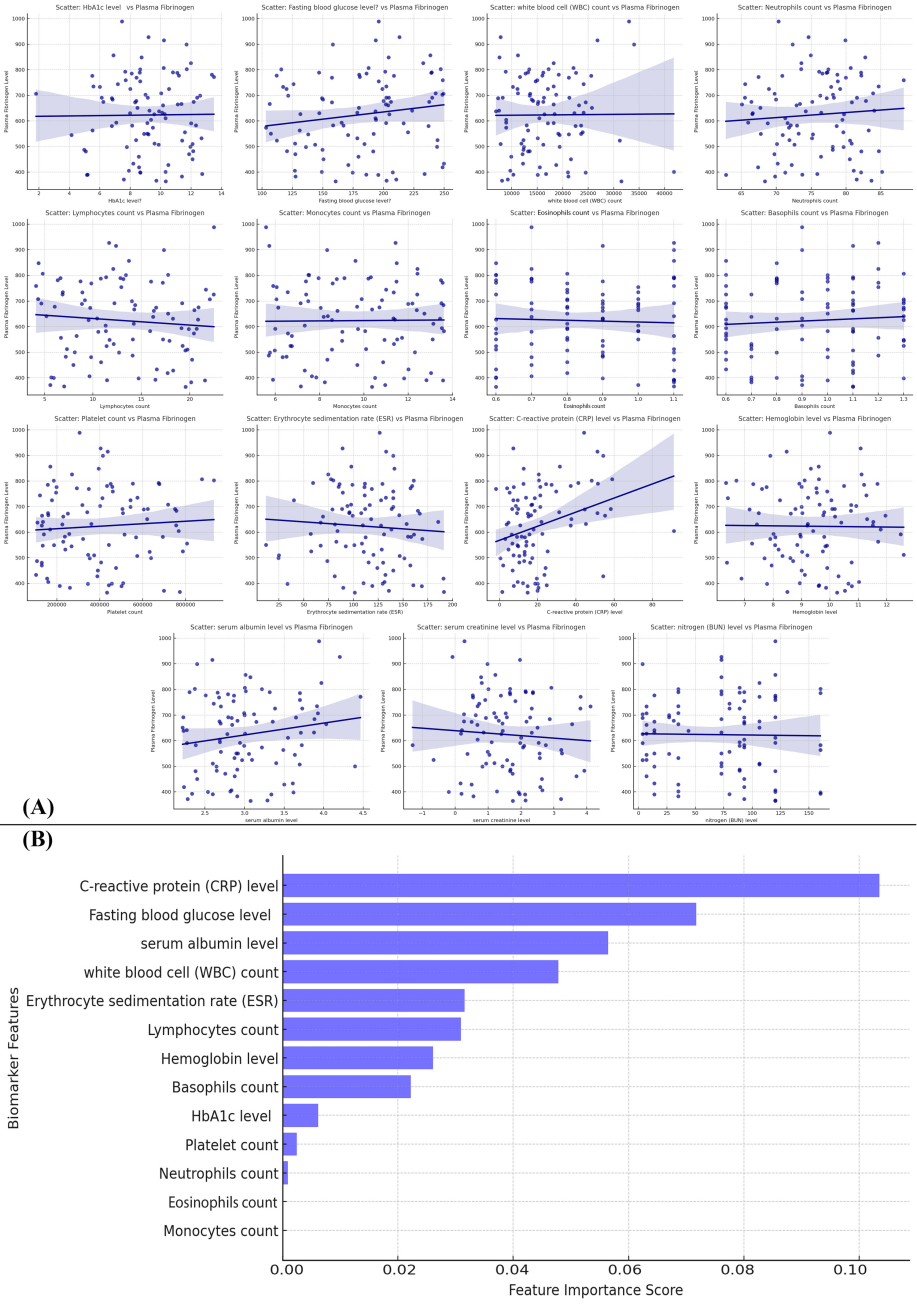
Key Biomarkers Influencing Plasma Fibrinogen Levels in Diabetic Foot Ulcer Patients: Scatter Plot and Decision Tree Analysis (A) Scatter plots illustrating the relationship between various biomarkers (e.g., HbA1c, fasting blood glucose, WBC count) and plasma fibrinogen levels. Some biomarkers show a stronger positive correlation with fibrinogen levels. (B) A bar chart ranking biomarkers by their feature importance in predicting plasma fibrinogen levels. C-reactive protein (CRP), fasting blood glucose, and serum albumin are identified as the most important biomarkers.

The figure presents (A) scatter plots analyzing the relationship between plasma fibrinogen levels and various biomarkers, and (B) a decision tree-based feature importance ranking for predicting fibrinogen levels. The scatter plots reveal that CRP exhibits the strongest positive correlation with fibrinogen, suggesting a significant role of inflammation in fibrinogen elevation. The decision tree analysis ranks CRP as the most important predictor of fibrinogen levels, with the highest feature importance score, followed by fasting blood glucose and serum albumin. These findings underscore the key role of systemic inflammation (CRP), metabolic dysregulation (glucose levels), and liver function (albumin) in regulating fibrinogen levels, indicating their potential as biomarkers for disease progression in DFU patients (Figure 15).

## Discussion

DFUs represent a major complication of diabetes, often leading to hospitalization, amputations, and increased mortality. The prevalence of DFUs is concerning, affecting approximately 15% to 25% of the diabetic population, with a significant proportion of these individuals experiencing recurrent ulcerations [16-18]. Understanding the factors contributing to DFU severity, progression, and healing is essential for developing effective management strategies. Recent studies have highlighted plasma fibrinogen levels as a potential biomarker for assessing DFU severity, vascular impairment, and infection risks, providing a novel approach to stratifying patients based on their risk [1,6,9].

Plasma fibrinogen is an acute-phase reactant involved in both coagulation and inflammation. Elevated fibrinogen levels have been linked to various chronic conditions, including diabetes [9,19]. Our study found a strong correlation between elevated fibrinogen levels and increased ulcer severity, as classified using the Wagner system. The statistical analysis supports the hypothesis that higher fibrinogen concentrations correlate with more advanced stages of DFU, with fibrinogen levels rising from Wagner Grade 2 ulcers (median 503.51 mg/dL) to Wagner Grade 5 ulcers (median 720.77 mg/dL) [6,5]. This trend highlights the relationship between fibrinogen elevation, severe tissue damage, and systemic inflammation that accompanies more advanced ulceration stages [1,19].

Moreover, our findings align with previous studies suggesting that hyperfibrinogenemia is a significant predictor of non-healing wounds and amputation risk in diabetic patients with foot ulcers [1,20]. Notably, patients with chronic wounds had significantly higher fibrinogen levels compared to those without ulcers [21]. This supports the idea that elevated fibrinogen not only reflects the inflammatory environment in DFUs but also serves as a marker for poor clinical outcomes, including hypercoagulable states and impaired healing mechanisms inherent in the diabetic population [6,20].

PAD is a major contributor to ulcer pathogenesis, primarily through its association with vascular impairment. Our study emphasizes the potential of fibrinogen as a biomarker for assessing PAD severity, demonstrated by significant differences in fibrinogen levels between moderate and severe vascular disease scenarios [19,21]. The correlation between fibrinogen and vascular function highlights its role in mediating blood viscosity and contributing to endothelial dysfunction key factors exacerbating both PAD and ulcer development [6,21]. The use of decision tree modeling further enhances the prognostic value of fibrinogen, identifying levels exceeding 531 mg/dL as predictive of severe vascular disease. These findings suggest the potential to integrate fibrinogen monitoring into routine vascular assessments alongside tools like the ABI and Doppler ultrasound [6,21].

The relationship between plasma fibrinogen levels and bacterial infection in DFUs is also crucial. Our results show that patients with infections caused by more virulent bacteria, such as Clostridium species and Klebsiella pneumoniae, had significantly higher fibrinogen levels compared to those infected with less aggressive organisms like Escherichia coli. This suggests that elevated fibrinogen levels may indicate more severe infections, potentially due to the inflammatory response triggered by these pathogens [1,22]. Previous studies have shown a robust association between hyperfibrinogenemia and increased cytokine activity, immune activation, and systemic inflammation, underscoring fibrinogen's relevance in predicting severe bacterial infections in DFUs [1,22].

In addition to ulcer severity and infection, we examined the correlation between fibrinogen and clinical parameters, uncovering significant relationships with CRP, fasting blood glucose, and serum albumin. CRP levels were identified as the strongest predictor of fibrinogen concentration, highlighting the intertwined nature of inflammation and glycemic control in the pathophysiology of DFUs [9,21]. Hyperglycemia is known to induce oxidative stress, exacerbating inflammation and fibrinogen synthesis in the liver, illustrating the interdependence of metabolic dysregulation and systemic inflammatory processes [23]. Additionally, moderate correlations were observed between fibrinogen and other inflammatory markers such as WBC counts, erythrocyte sedimentation rates, and lymphocyte counts, reflecting the broader immune response contributing to fibrinogen elevation in DFUs [23].

Clinically, these findings have significant implications for DFU management, particularly in the context of fibrinogen's role in risk stratification and treatment planning. Given the established correlations between fibrinogen levels and variables indicating ulcer severity, vascular health, and infection, monitoring plasma fibrinogen could facilitate the categorization of patients into distinct risk groups. This stratification would enable more tailored treatment approaches, including more aggressive management for those with elevated fibrinogen and increased vascular compromise [6,19]. Elevated fibrinogen levels not only suggest a greater risk of complications but also signal the need for stricter glycemic control, recognizing that poor glucose management contributes to elevated fibrinogen and impairs healing prospects [23].

The potential for future research to explore fibrinogen-targeted therapies is also an exciting area for investigation. Studies examining the efficacy of anticoagulant or anti-inflammatory treatments specifically designed to lower fibrinogen levels could significantly improve healing rates and reduce complications in diabetic patients with DFUs [18,24]. Furthermore, longitudinal studies tracking fibrinogen levels and correlating them with ulcer healing outcomes, amputation rates, and patient mortality could provide critical evidence supporting the routine use of fibrinogen as a biomarker in diabetic foot care [6,25].

This study has several limitations that should be considered when interpreting the findings. Firstly, the cross-sectional design of the study means that it cannot establish causality between elevated fibrinogen levels and the severity of DFUs. While a correlation between fibrinogen and DFU severity was found, it is not possible to determine whether elevated fibrinogen is a cause or a consequence of ulcer progression. Secondly, the sample size used in the study may be relatively small, which could limit the generalizability of the results to a larger, more diverse diabetic population. The study was conducted at a single center, which introduces the possibility of local biases related to patient characteristics, clinical practices, and demographic factors, thus reducing the external validity of the findings. Furthermore, the inclusion of only patients with active foot ulcers may have led to selection bias, excluding individuals with healed ulcers or those who may be in the early stages of ulcer development. The lack of a healthy control group also limits the ability to determine whether elevated fibrinogen levels are specific to DFUs or are simply a general marker of systemic inflammation in diabetic patients. The study did not include diabetic patients without DFUs or healthy individuals, which would have helped clarify whether fibrinogen elevation is DFU-specific or a broader marker of inflammation. Additionally, while the study aimed to examine fibrinogen as a biomarker for DFU severity, we recognize that a control group would strengthen the findings and help differentiate fibrinogen's role in DFUs from other inflammatory conditions. Moreover, the use of the Wagner system to classify ulcer severity introduces some variability in the assessment, as the Wagner system is subjective and may vary between clinicians. The presence of comorbidities such as cardiovascular disease, kidney disease, or other chronic conditions in the study population may have confounded the results, as these conditions could independently influence fibrinogen levels and ulcer severity. Another limitation is that the study did not systematically assess the severity or type of bacterial infections in the DFUs, which could influence fibrinogen levels. Some patients may have received medications such as anti-inflammatory drugs or anticoagulants, which could also have affected their fibrinogen levels, but these variables were not controlled for in the analysis. Additionally, the study did not record medication history, such as the use of insulin or metformin, which can significantly affect wound healing. Medications like insulin and metformin, especially when combined, have been shown to improve wound healing and may influence the interpretation of the findings. Finally, the timing of data collection is a potential limitation, as fibrinogen levels can fluctuate depending on the stage of disease or treatment. For example, fibrinogen may be elevated during acute inflammation but may not accurately reflect the long-term status of the wound or the patient’s overall health. Furthermore, health, hygiene, and lifestyle factors, such as higher income leading to better living standards, could also impact foot ulcer resolution but were not considered in this study. These factors should be considered when interpreting the study’s conclusions and assessing the potential clinical applications of fibrinogen as a biomarker for DFU severity. Additionally, as the study is cross-sectional, causality cannot be established, and future research incorporating a control group and longitudinal follow-up would be valuable to strengthen the claim that fibrinogen is a reliable biomarker for DFU risk stratification.

## Conclusions

The results of this study strongly indicate that elevated plasma fibrinogen levels could serve as an effective biomarker for assessing the severity of DFUs in patients with diabetes. By establishing a clear correlation between fibrinogen levels and factors such as ulcer severity, vascular health, and the risk of infection, fibrinogen monitoring holds promise for enhancing risk stratification in diabetic foot care. This approach could facilitate more personalized and targeted treatment plans, allowing clinicians to identify patients at higher risk for complications such as non-healing wounds or amputation. Moreover, the ability to monitor fibrinogen levels could provide valuable insights into the effectiveness of ongoing treatment and guide adjustments in therapeutic strategies. Given its potential as a predictive and diagnostic tool, future research should aim to validate fibrinogen as a standard marker in the management of DFUs.
